# Lignans Associated Differences in Salt Stress Responses of Flax (*Linum usitatissimum* L.) Genotypes In Vitro

**DOI:** 10.3390/cells15090796

**Published:** 2026-04-28

**Authors:** Moumita Roy Chowdhury, Katarína Ražná, Jindra Valentová, Emil Švajdlenka, Eva Ivanišová, Anirban Jyoti Debnath, Jit Mukherjee, Veronika Štefúnová, Mizgin Mehmet, Paračková Patrícia, Marián Miko

**Affiliations:** 1 Institute of Plant and Environmental Sciences, Faculty of Agrobiology and Food Resources, Slovak University of Agriculture in Nitra, Trieda Andreja Hlinku 2, 949 76 Nitra, Slovakia; veronika.stefunova@uniag.sk (V.Š.); marian.miko@uniag.sk (M.M.); 2 Department of Chemical Theory of Drugs, Faculty of Pharmacy, Comenius University Bratislava, Odbojárov 10, 832 32 Bratislava, Slovakia; valentova@fpharm.uniba.sk (J.V.); svadlenka@fpharm.uniba.sk (E.Š.); patricia.parackova@fpharm.uniba.sk (P.P.); 3 Institute of Food Sciences, Faculty of Biotechnology and Food Sciences, Slovak University of Agriculture in Nitra, Trieda Andreja Hlinku 2, 949 76 Nitra, Slovakia; eva.ivanisova@uniag.sk; 4 Institute of Chemistry and Environmental Science, Faculty of Natural Sciences, University of Ss. Cyril and Methodius in Trnava, Námestie Jozefa Herdu 2, 917 01 Trnava, Slovakia; anirbandebnath@ymail.com; 5 Department of Computer Science and Engineering, Birla Institute of Technology, Mesra 835215, India; jit.mukherjee@bitmesra.ac.in; 6 Department of Field Crops, Faculty of Agriculture, Ege University, Bornova, 35100 Izmir, Türkiye; mehmetmizgin36@gmail.com

**Keywords:** salt stress, flax, elicitation in vitro, lignan, antioxidant analysis, microRNA

## Abstract

**Highlights:**

**What are the main findings?**

- Salinity modulates SDG, SECO, PINO, MATA and LARI in flax genotypes and salinity stress induced de novo synthesis of PINO derivatives.
- The Agram genotype showed consistently higher antioxidant-related parameters, while CDC Bethune exhibited distinct lignan compositional responses. Along with that observed miRNA expression patterns suggest a potential regulatory involvement.

**What are the implications of the main findings?**

- Lignans are associated with the response to salinity stress in flax.
- The defense responses to salinity stress in flax are strongly genotype dependent.

**Abstract:**

The objective of this study was to investigate the association between lignan content and stress responses in flax genotypes with contrasting lignan levels. For this purpose, two flax (*Linum usitatissimum* L.) genotypes, Agram and CDC Bethune, were selected based on their differing lignan profiles. We quantified secoisolariciresinol diglucoside, pinoresinol, pinoresinol diglucoside, matairesinol, and lariciresinol in both control and salt-stressed plants. In parallel, antioxidant activity, flavonoid, polyphenols, and phenolic acid content were determined to assess the overall antioxidant potential and phenolic response under saline conditions. The Agram genotype appears to activate defense mechanisms that enhance antioxidant capacity, which is largely mediated by polyphenolic compounds and distinct patterns of microRNA regulation. By contrast, the CDC Bethune genotype primarily responds to salinity stress by inducing lignan biosynthesis. Differential lignan modulation, contrasting antioxidants and miRNA profiles, shows substantial intergenotypic differences in how flax activates distinct defense pathways.

## 1. Introduction

Soil salinity represents one of the most significant constraints to agricultural productivity. Salinity affects nearly 1381 million hectares (Mha), or 10.7 percent of the total global land area, including 10% of rainfed cropland and about 10% of irrigated cropland [1]. Salinity stress adversely affects multiple stages of the plant life cycle in glycophyte crops, including seed germination, vegetative growth, development, flowering, and fruiting [2]. Elevated soil salinity limits water and nutrient uptake, inducing physiological drought and disrupting cellular osmotic balance [3]. To re-establish osmotic homeostasis, plants reallocate photosynthates toward the synthesis of compatible osmolytes such as proline and glycine betaine [4]. This metabolic reprogramming, however, comes at the expense of photosynthetic efficiency and biomass accumulation under saline conditions. Consequently, stress-induced growth arrest is considered an adaptive response that conserves carbohydrates for basal metabolism and sustained energy supply, thereby supporting recovery following stress alleviation [5].

In addition to osmotic stress, salinity disrupts cellular energy homeostasis by enhancing electron leakage from photosynthetic, respiratory, and photorespiratory electron transport chains in chloroplasts, mitochondria, and peroxisomes, respectively. The leakage of electrons results in excessive generation of reactive oxygen species (ROS), causing oxidative damage to lipids, proteins, carbohydrates, and nucleic acids, and ultimately leading to cellular dysfunction and death [6,7]. To mitigate oxidative injury, plants activate a coordinated antioxidant defense system comprising enzymatic, non-enzymatic, and non-protein amino acid components [6,8]. Among non-enzymatic antioxidants, polyphenols—including phenolic acids, flavonoids, stilbenoids, and lignans—are particularly effective ROS scavengers due to the presence of multiple hydroxyl groups in their molecular structures [9].

Lignans are dimeric phenylpropanoid secondary metabolites that are widely distributed in higher plants, where they play a crucial role in plant defense. Structurally, lignans are formed by β–β′ coupling between two phenylpropane units at carbon 8 [10]. Naturally occurring lignans display considerable structural diversity, arising from various substitution patterns and degrees of modification [11,12]. Lignan biosynthesis is markedly induced under abiotic stress conditions, including drought, salinity, temperature extremes, heavy metal exposure, and ultraviolet radiation—largely due to their strong antioxidant properties [13,14]. Accordingly, stress-tolerant genotypes frequently accumulate higher levels of lignans, as demonstrated in drought-tolerant sesame [15] and cadmium-tolerant rapeseed genotypes [16]. Beyond their role in abiotic stress tolerance, lignans also exhibit antibacterial, antifungal, and antifeedant activities, further contributing to plant defense mechanisms [17].

MicroRNAs (miRNAs) are a class of endogenous, non-coding RNA molecules approximately 19–24 nucleotides in length that negatively regulate gene expression at the post-transcriptional level. In plants, miRNAs are incorporated into the RNA-induced silencing complex, in which the Argonaute protein facilitates sequence-specific binding to target messenger RNAs, leading to mRNA cleavage and subsequent gene silencing [18]. Since the first discovery of plant miRNAs by Reinhart et al., these regulatory molecules have been recognized as key modulators of a wide range of physiological and developmental processes [19]. Accumulating evidence further demonstrates that miRNAs play critical roles in plant responses to abiotic stresses, including salinity. Notably, several conserved miRNAs—miR156, miR159, miR160, miR164, miR166, miR167, miR168, miR169, miR172, miR319, miR394, miR395, miR396, miR397, miR399, and miR408—have been identified as integral components of plant salt-stress responses [20]. These miRNAs exert their functions primarily through the regulation of hormone signaling pathways [21] and antioxidant defense systems [22].

Flax (*Linum usitatissimum* L.; family *Linaceae*), commonly referred to as flaxseed or linseed, is a multipurpose crop cultivated for food, oil, and fiber, and is recognized as one of the richest plant sources of lignans. The major lignans present in flax include secoisolariciresinol (SECO), secoisolariciresinol diglucoside (SDG), lariciresinol (LARI), matairesinol (MATA), pinoresinol (PINO) and pinoresinol diglucoside (PINO-DG). Importantly, lignan content exhibits substantial variation among flax genotypes [23,24]. Despite its considerable nutritional and industrial value, flax is highly sensitive to abiotic stresses, including drought, salinity, and heat, which severely compromise plant growth, survival, and productivity [25]. Owing to its exceptionally high lignan content and pronounced susceptibility to salinity stress, flax constitutes an excellent model system for elucidating the role of lignans in plant responses to salinity stress.

We investigated the relationship between lignan content and salinity stress responses in flax using genotypes with contrasting lignan levels. We hypothesized that high salinity elicits genotype-specific alterations in the composition and abundance of individual lignan compounds, with these stress-induced responses operating through coordinated antioxidant and miRNA regulatory mechanisms, indicative of metabolite-level adaptation to salinity stress. For this purpose, two flax genotypes, Agram and CDC Bethune, were selected based on their differing lignan profiles, with Agram showing double the SECO values. We quantified SDG, SECO, PINO, PINO-DG, MATA, and LARI in both control and salt-stressed plants using High-Performance Liquid Chromatography (HPLC) and Mass Spectrometry (MS). Within this framework, three de novo synthesized PINO derivatives were identified under stress conditions. Our data show that flax defense responses to salinity stress are genotype-dependent, reflecting distinct stress-responsive defense strategies within a single species.

## 2. Materials and Methods

### 2.1. Plant Materials and Stress Treatment

*L. usitatissimum* seeds of the cultivars Agram and CDC Bethune used in the experiments were provided by the Research and Breeding Station Vigľaš-Pstruša, National Agricultural and Food Centre, Research Institute of Plant Production, Slovakia. The information about the SECO content of both cultivars was provided by the Agritec Plant Research Ltd. (Zemědělská, Šumperk, Czech Republic). For surface sterilization, seeds were rinsed in distilled water for 5 min, followed by immersion in 70% (*v*/*v*) ethanol for 5 min, and then treated with 10% sodium hypochlorite (NaOCl) containing Tween-20 for 5 min. Seeds were subsequently rinsed three times with distilled water for 5 min each. Sterilized seeds were cultivated under aseptic conditions in sterile plastic boxes with hermetic covers and breathable strips (Duchefa Biochemie, Haarlem, The Netherlands). Cultivation was conducted on Murashige and Skoog (MS) medium supplemented with macro- and micronutrients, vitamins (Duchefa Biochemie, The Netherlands), and Plant Preservative Mixture (Plant Cell Technology, Smithfield, UT, USA), serving as the control medium. For salt stress treatment, the cultivation medium was supplemented with 200 mM NaCl. For each genotype (Agram and CDC Bethune), five vessels containing 30 seeds each were cultivated per treatment (control and stress) under in vitro conditions in a growth chamber maintained at 22 °C, with a 16/8 h light/dark photoperiod and a light intensity of 6500 lux. Seedlings were monitored daily. The biological material was divided into the quantities required for the subsequent analyses.

The experimental design sought to create conditions conducive to the reliable detection of stress-induced alterations in lignan biosynthesis, while considering the inherently different basal levels of secoisolariciresinol (SECO) in the seeds of the examined flax genotypes. In this context, the salt concentration was selected not to reflect average field conditions, but rather to serve as a controlled, acute stress signal that would elicit measurable metabolic responses. In the absence of a non-stress germination phase and due to the direct exposure of seeds to saline conditions, salt-treated plants were harvested four weeks after the start of the experiment, whereas control plants were collected two weeks after sowing.

### 2.2. Lignan Content

#### 2.2.1. Sample Preparation for HPLC Analysis

Lignans were extracted from flax seedlings using a modified method according to the following method mentioned in Popova et al. [26]. The flax seedlings were homogenized under dry nitrogen, and 100 mg of homogenate was diluted in 10 mL of 80% (*v*/*v*) methanol. The solutions were vortexed for 1 min and sonicated for 60 min (Ultrasonic bath ELMASONIC S15H), followed by centrifugation (SIGMA 3-16K) at 5500 rpm for 15 min. The supernatants were decanted and used for analysis.

#### 2.2.2. HPLC-UV-MS/MS Analysis

HPLC-UV-MS/MS analysis was used to evaluate the content of SDG, SECO, LARI, MATA, PINO, and PINO-DG in flax seedlings. The lignan standards were purchased from TargetMol (Boston, MA, USA) and MedChemExpress (Monmouth Junction, Middlesex, NJ, USA). HPLC analysis was performed using an Agilent 1260 HPLC system (Agilent Technologies, Santa Clara, CA, USA) equipped with a diode array detector (DAD-UV) and coupled to a triple-quadrupole MS system, TRIPLE QUAD 3500 (AB SCIEX, Waltham, MA, USA) with an ESI source (AB SCIEX, MA, USA). Chromatographic separation of lignans was carried out using a Poroshell 120 EC-C18 column (150 mm × 4.6 mm, 2.7 μm) with a guard column (5 mm × 4.6 mm, 2.7 μm) (Agilent Technologies, CA, USA). The column temperature was maintained at 30 °C, and the injection volume was 1–5 μL. The mobile phase consisted of 1 mM HCOONH_4_ and 0.1% HCOOH in methanol (MFA) and 1 mM HCOONH_4_ with 0.1% HCOOH in water (MFB). Lignans were analyzed under the following gradient elution conditions at a flow rate of 0.3 mL/min: the gradient was initiated at 30% MFA/70% MFB (0 min), followed by a linear increase to 100% MFA/0% MFB at 14 min, which was maintained from 14 to 16 min. The mobile phase composition was then returned to 30% MFA/70% MFB at 18.1 min and maintained at this initial condition until 25 min to allow for column re-equilibration.

Mass spectra were acquired in the negative ionization mode using an optimized ionization potential of −4500 V. The ion source temperature was set to 450 °C, with a nebulizer gas (air) flow rate of 14 L min^−1^. Samples were analyzed in duplicate, with two independently prepared and parallel-analyzed extracts derived from the same biological sample to assess analytical variability. Calibration curves were generated using authentic standards (PINO-Dg, SECO-Dg, LARI, SECO, PINO, and MATA) to establish instrument response and enable quantitative evaluation (µg × g^−1^ fresh weight).

Quantification was performed using multiple reaction monitoring (MRM) to confirm analyte identity. The consistency of signal response for each compound was assessed using the relative standard deviation (RSD, %), which reflects the deviation between quantitative values obtained from the first and second MRM transitions and thus indicates the reliability of analyte identification and quantification. In addition, UV spectra were recorded over the range of 190–400 nm to monitor co-eluting peaks and to minimize potential ion suppression or enhancement effects.

Data from HPLC–UV–MS/MS analyses were acquired and processed using Analyst® 1.7 software for the Agilent 1260 HPLC system (Agilent Technologies, CA, USA), with subsequent evaluation performed in Microsoft Excel.

### 2.3. Biochemical Analysis

#### 2.3.1. Sample Preparation

One gram of the sample was extracted with 10 mL of 80% ethanol for 2 h. The extract was then centrifuged at 4000× *g* for 10 min using a Rotofix 32 A centrifuge (Hettich, Vlotho, Germany). The obtained supernatant was collected and subjected to analyses of antioxidant activity, total polyphenols, total flavonoids, and total phenolic acids.

#### 2.3.2. Antioxidant Activity Determination—DPPH Method

Antioxidant activity was determined using the 2,2-diphenyl-1-picrylhydrazyl (DPPH) assay, following the method described by Sánchez-Moreno et al. [27]. The DPPH solution was prepared by dissolving 0.025 g of DPPH in 100 mL of ethanol. Subsequently, 4 mL of this solution was mixed with 1 mL of the sample extract. Absorbance was measured at 515 nm using a Jenway spectrophotometer (6405 UV/Vis, Nottingham, UK). The free radical scavenging capacity of the samples was expressed as mg of Trolox equivalent antioxidant capacity (TEAC) per gram of sample, based on a Trolox calibration curve (10–100 mg/L; R^2^ = 0.989).

#### 2.3.3. Total Polyphenol Content

Total polyphenol content (TPC) was determined spectrophotometrically using a modified Folin–Ciocalteu method as described by Singleton and Rossi [28]. An aliquot of 0.1 mL of the sample was mixed with 0.1 mL of Folin–Ciocalteu reagent, 1 mL of 20% sodium carbonate, and 8.8 mL of distilled water. After incubation in the dark for 30 min, absorbance was measured at 700 nm using a Jenway spectrophotometer (6405 UV/Vis, Nottingham, UK). Results were expressed as mg gallic acid equivalents (GAE) per gram of sample, based on a gallic acid calibration curve (25–300 mg/L; R^2^ = 0.999).

#### 2.3.4. Total Phenolic Acid Content

Total phenolic acid content (TPAC) was determined using the method described by Jain et al. [29]. A 0.5 mL aliquot of the sample extract was mixed with 0.5 mL of 0.5 M hydrochloric acid, 0.5 mL of Arnova reagent (10% NaNO_2_ + 10% Na_2_MoO_4_), 0.5 mL of 1 M sodium hydroxide (*w*/*v*), and 0.5 mL of distilled water. Absorbance was measured at 490 nm using a Jenway spectrophotometer (6405 UV/Vis, Nottingham, UK). Caffeic acid was used as the standard, and results were expressed as mg caffeic acid equivalents (CAE) per gram of sample, based on a calibration curve (1–200 mg/L; R^2^ = 0.9997).

#### 2.3.5. Total Flavonoid Content

Total flavonoid content (TFC) was determined using a modified method described by Willett (2002) [30]. A 0.5 mL aliquot of the sample was mixed with 0.1 mL of a 10% (*w*/*v*) ethanolic aluminum chloride solution, 0.1 mL of 1 M potassium acetate, and 4.3 mL of distilled water. After incubation in the dark for 30 min, absorbance was measured at 415 nm using a Jenway spectrophotometer (6405 UV/Vis, Nottingham, UK). Quercetin was used as the standard, and results were expressed as mg quercetin equivalents (QEs) per gram of sample, based on a calibration curve (1–400 mg/L; R^2^ = 0.9996).

### 2.4. Molecular Analysis

#### 2.4.1. Small RNA Isolation

Small RNA (sRNA) was isolated from 30 mg of seedling tissue using the NucleoSpin miRNA isolation kit (Macherey-Nagel, Allentown, PA, USA) according to the manufacturer’s instructions. RNA concentration and purity were assessed using Implen NanoPhotometer^®^, and the isolated RNA was stored at −20 °C until further use.

#### 2.4.2. Two-Tailed RT-qPCR (TT-qPCR) Assay for miRNA

miRNA expression analysis was performed using the two-tailed reverse transcription quantitative PCR (TT-qPCR) assay (BioVendor—Laboratorní medicína a.s., Brno, Czech Republic), following the protocol described by Androvic et al. [31]. The assay is designed for the quantification of miRNA expression using miRNA-specific structured primers for reverse transcription, followed by SYBR Green-based qPCR. The assay includes all necessary reagents, including specific primers for each miRNA, for the analysis of samples in triplicate. The sequences of the two miRNAs (lus-miR168a and lus-miR396) were provided to BioVendor, based on flax miRNA sequences provided by the miRBase database (https://www.mirbase.org/) (accessed on 2 April 2022), and the corresponding reverse transcription primers, as well as the forward and reverse PCR primers, were supplied as part of the assay kit. U6 small nuclear RNA (snRNA) was used as the endogenous control for normalization (U6 snRNA Forward primer: 5′–GACATCCGATAAAATTGGAACG–3′, Reverse primer: 5′–TTGGACCATTTCTCGATTTGTG–3′).

At first, isolated sRNA (NucleoSpin miRNA, Macherey Nagel) was reverse-transcribed into cDNA using miRNA-specific RT primers on a C1000 Thermal Cycler (Bio-Rad Laboratories, Inc., Hercules, CA, USA). The reaction mixture, containing sRNA, RT mix, RT primer, RT enzyme, and nuclease-free water (NFW), was incubated at 25 °C for 5 min, followed by 50 °C for 15 min and 85 °C for 5 min. The resulting cDNA was diluted by the addition of nuclease-free water (NFW) and subsequently subjected to qPCR analysis using a CFX96 Real-Time PCR System (Bio-Rad). The PCR reaction mixture consisted of 2× PCR mix, 1 μM of each gene-specific forward and reverse primer, cDNA, and NFW. Thermal cycling conditions included an initial incubation at 95 °C for 30 s, followed by 40 cycles of denaturation at 95 °C for 5 s, annealing at 60 °C for 15 s, and extension at 72 °C for 10 s. The individual procedures were carried out in accordance with the provider’s instructions. Amplicon specificity was confirmed by melting curve analysis. Gene expression levels were computed relative to the expression of the endogenous control gene under the same conditions using the 2^–∆∆Ct^ method [32].

### 2.5. Statistical Analysis

All biochemical and molecular data were normalized prior to multivariate visualization. Biochemical analyses were performed using two biological replicates, which were analyzed individually. In contrast, for miRNA expression analysis, three biological replicates were pooled prior to RNA extraction, and each pooled sample was subsequently analyzed by qPCR using three technical replicates. Radar plots were generated to compare the relative stress-response profiles of the two genotypes (Agram and CDC Bethune) across twelve biochemical and molecular parameters, using normalized values to allow direct comparison among traits. Stress index values were calculated and visualized using bar diagrams to assess genotype- and trait-specific responses to salinity stress. For heatmap analysis, Z-score normalization was applied to each variable (Z = (x − μ)/σ) to standardize data across genotypes and highlight relative induction or suppression patterns. Hierarchical clustering based on Z-scores was used to identify genotype-dependent response groupings. All statistical analyses and visualizations were performed using Matplotlib and Python, and differences were considered statistically significant at *p* < 0.05.

## 3. Results

### 3.1. Effect of Salinity Stress on Plant Morphology/In Vitro Growth of Seedlings

To enable reliable detection of stress-induced changes in lignan compositional dynamics, the experiment was designed to account for the intrinsically different basal levels of secoisolariciresinol (SECO) present in the seeds of the studied flax genotypes. Accordingly, salinity stress was imposed as a controlled, acute stimulus to provoke measurable metabolic responses in the context of molecular and biochemical parameters under in vitro conditions rather than replicating average field environments. Another aspect of the experimental design was the direct exposure of seeds to saline conditions immediately upon sowing, without a preceding non-stress germination phase. Although we recognize that this approach does not fully replicate typical field conditions, it reflects scenarios in which seeds encounter salinity at the earliest stages of development.

**Figure 1:**
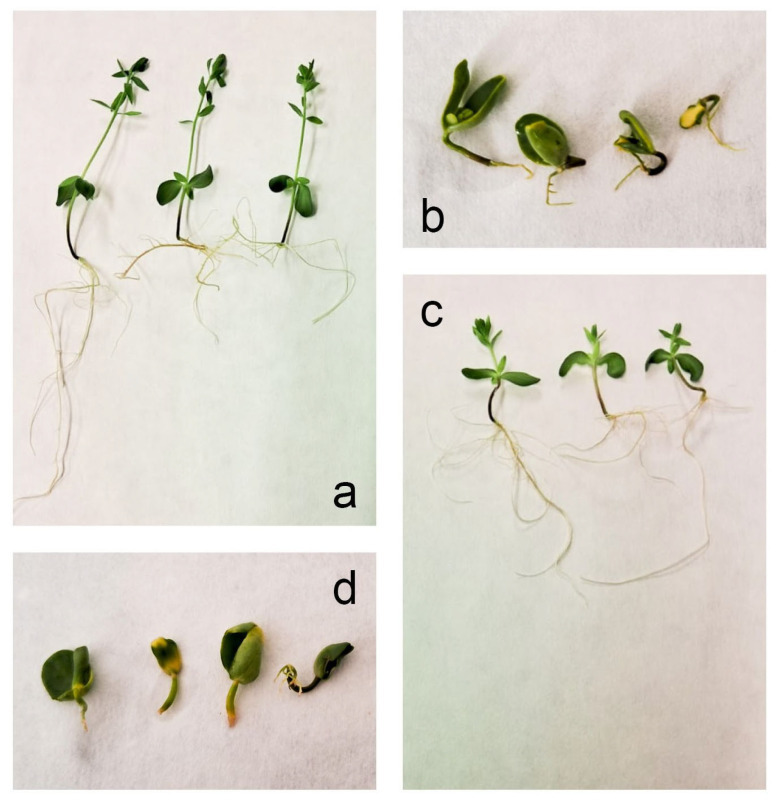
Changes in morphological parameters of two flax genotypes, Agram and CDC Bethune, after 4 weeks of 200 mM NaCl stress. (**a**) Agram control plants, (**b**) Agram stressed plants, (**c**) CDC Bethune control plants, (**d**) CDC Bethune stressed plants.

Plants exposed to 200 mM NaCl showed severely inhibited growth; therefore, they were maintained for 30 days prior to harvest. Control plants exhibited normal growth within 15 days of cultivation and were harvested. Genotype-specific differences in lignan content were not accompanied by changes in growth under either control or salinity stress. However, differential responses to salinity stress were evident from phenotypic observations. Control plants of Agram (Figure 1a) and CDC Bethune (Figure 1c) displayed healthy growth, whereas NaCl-stressed plants of Agram (Figure 1b) and CDC Bethune (Figure 1d) showed a marked reduction in growth compared to their respective controls.

### 3.2. Genotype-Specific Modulation of Lignan Profiles Under Salinity Stress

**Figure 2:**
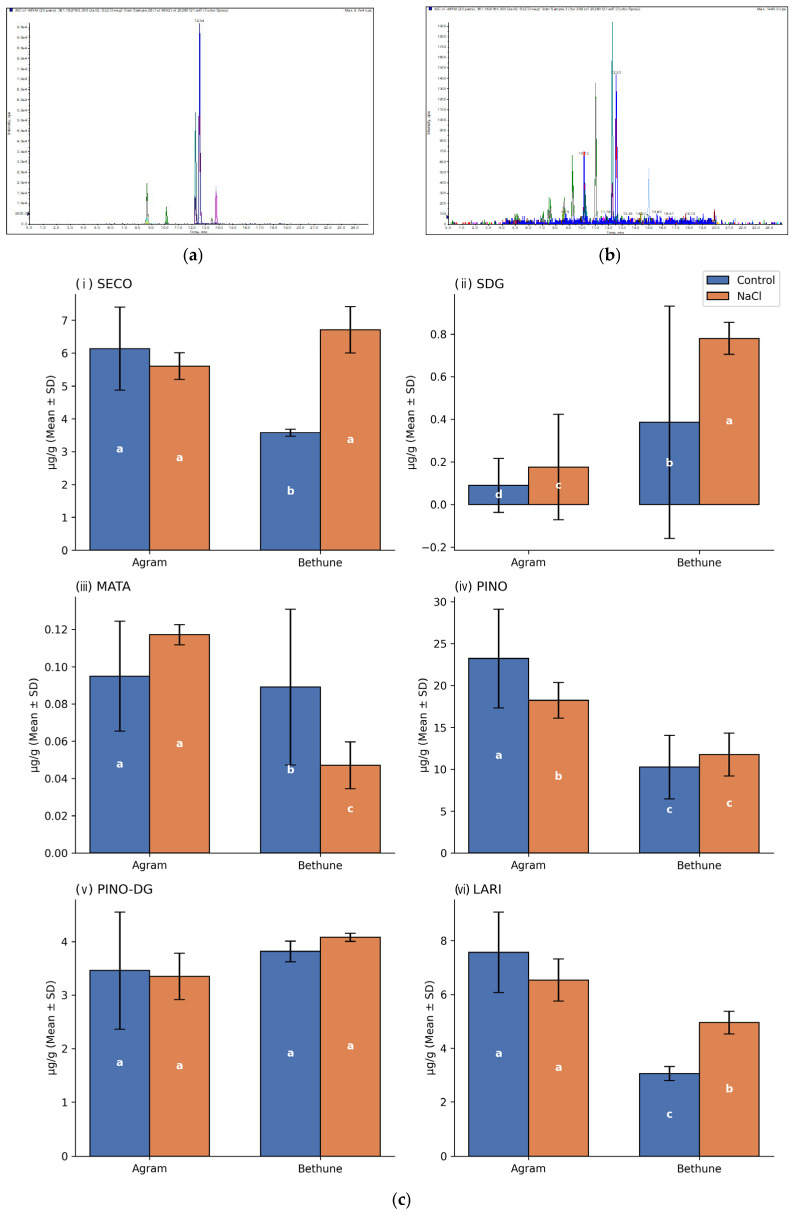
Effect of NaCl stress on lignan content in Agram and Bethune. Error bars represent standard deviation (SD). (**a**) HPLC-MS/MS of 0.2 ul of standards mix (PDG-8.66, LARI, SECO, PINO-13.43, MATA in retention order) (**b**) HPLC-MS/MS of 5 ul CDC Bethune extract (PDG-8.64, PINO der.1-9.27, SDG-10.17, PINO der.2-10.17, PINO der.3-10.99, LARI, SECO, PINO in retention order). (**c**) Relative levels of SECO (**i**), SDG (**ii**), PINO (**iv**), PINO-DG (**v**), MATA (**iii**), and LARI (**vi**) in control and 200 mM NaCl-treated plants are shown. Distinct letters denote statistically significant differences at *p* < 0.05, based on the applied statistical analysis.

The quantified concentrations of individual lignans are summarized in Table 1. In Agram, SDG content increased numerically under NaCl stress; however, this difference was not statistically significant compared with control plants. Although aglycone SECO levels were slightly lower after NaCl exposure than in control plants, the observed difference did not reach statistical significance. In contrast, CDC Bethune exhibited a significant increase in both SECO and SDG under NaCl stress. Specifically, NaCl treatment resulted in an approximately two-fold increase in SDG levels, while SECO content was also increased to about two-fold relative to control plants. The concentration of PINO-DG showed only minor variation between control and NaCl-treated plants in both genotypes. In Agram, the aglycone PINO accumulated at significantly higher levels in control plants compared with NaCl-stressed plants, whereas in CDC Bethune, differences in PINO content between treatments were small. Notably, PINO was the most abundant lignan in both genotypes, accumulating at levels several times higher than those of the other quantified lignans (Figure 2). The content of LARI was slightly higher in Agram control plants than in NaCl-treated plants, whereas in CDC Bethune, LARI levels were higher under NaCl stress than in control conditions. The concentration of MATA showed only minor variation in Agram, with slightly higher levels detected under NaCl stress. In CDC Bethune, only trace amounts of MATA were detected, and differences between treatments were negligible.

We observed in Agram and CDC Bethune plants three derivatives of PINO at concentrations higher than PINO and PDG (these three derivatives have retention times between PDG and PINO, and in MS, they have the same MRM transitions as PINO). PINO derivative 1 content was more than 10 times lower in NaCl-stressed CDC Bethune plants compared with their respective controls, whereas in Agram plants it was only half of its respective controls. Similar genotype-dependent patterns were observed for PINO derivative 2. Conversely, the accumulation of PINO derivative 3 was independent of both plant genotype and NaCl-induced salinity stress.

### 3.3. Antioxidant Responses Under Salinity Stress

**Figure 3:**
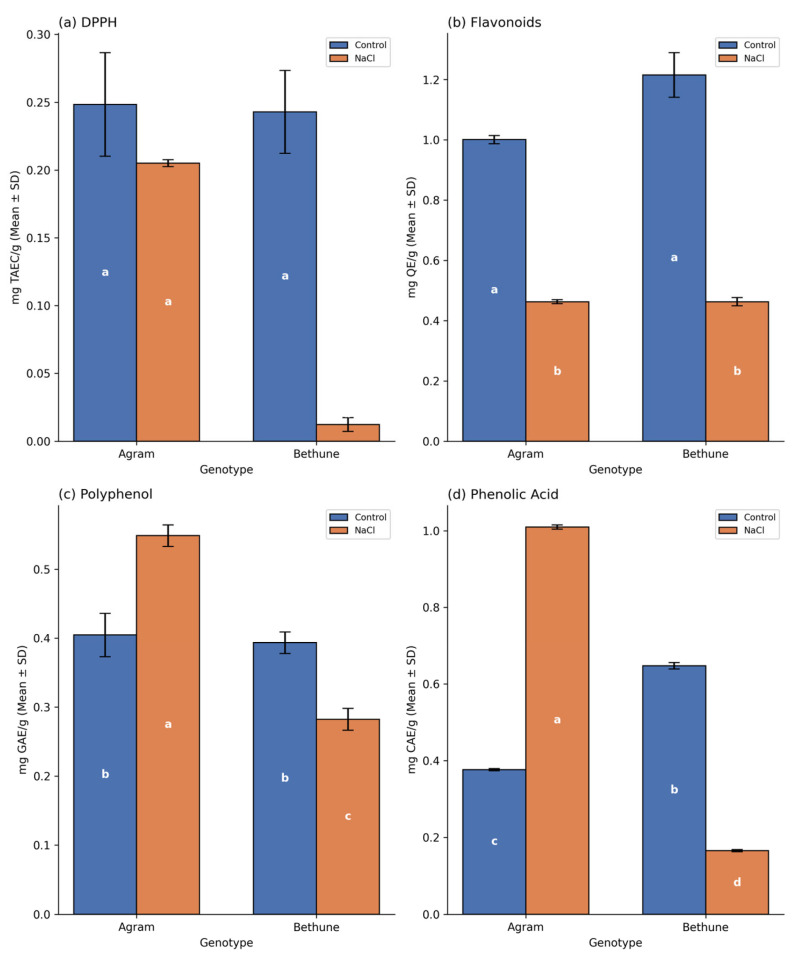
Total radical-scavenging activity and biochemical estimation of non-enzymatic antioxidants in two flax genotypes, Agram and Bethune, under control and 200 mM NaCl treatments. (**a**) Total radical-scavenging activity was determined using the DPPH assay. Levels of non-enzymatic antioxidants, including (**b**) flavonoids, (**c**) polyphenols, and (**d**) phenolic acids, were quantified. Error bars represent standard deviation (SD). Distinct letters denote statistically significant differences at *p* < 0.05, based on the applied statistical analysis.

Cellular stress activates antioxidant defense mechanisms in plants. In this study, salt stress induced significant genotype-dependent alterations in several non-enzymatic antioxidant markers in the flax genotypes Agram and CDC Bethune. The total radical-scavenging activity of antioxidants, as assessed by the DPPH assay, was reduced in Agram and showed a significant reduction in CDC Bethune under salt stress (Figure 3a). Similarly, salt stress resulted in a significant decline in TFC in both Agram and CDC Bethune genotypes (Figure 3b). In contrast, salt stress markedly increased the levels of TPC and TPAC in Agram, whereas these markers decreased in CDC Bethune (Figure 3c,d).

### 3.4. Regulation of Lignan-Associated miRNAs Under Salinity Stress

**Figure 4:**
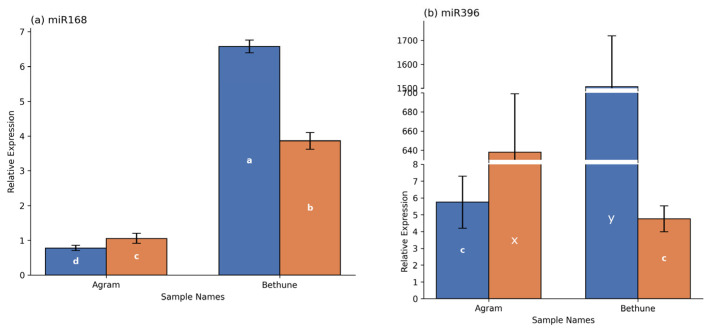
Expression analysis of lignan-biosynthetic miRNAs in two flax genotypes, Agram and CDC Bethune, under control and 200 mM NaCl treatments. The relative expressions of two miRNAs (**a**) miR168a and (**b**) miR396 were measured using the two-tailed qPCR assay kit using U6 snRNA as internal controls for normalization. Error bars represent standard deviation (SD). Distinct letters denote statistically significant differences at *p* < 0.05, based on the applied statistical analysis.

TT-qPCR analysis was performed to evaluate the expression of lus-miR168a and lus-miR396 under control and NaCl-stressed conditions. For lus-miR168a, expression was higher in Agram stressed plants compared with controls, whereas in CDC Bethune, expression was significantly higher in control plants than in stressed plants. Comparative analysis under salinity stress revealed that lus-miR168a expression was significantly higher in CDC Bethune than in Agram (Figure 4a). For lus-miR396, expression was significantly upregulated in NaCl-stressed Agram plants relative to controls, while in CDC Bethune, expression was higher in control plants than in stressed plants. Comparative analysis under salinity stress indicated that miR396 expression was significantly higher in Agram compared with CDC Bethune (Figure 4b).

### 3.5. Findings from Statistical Analysis

**Figure 5:**
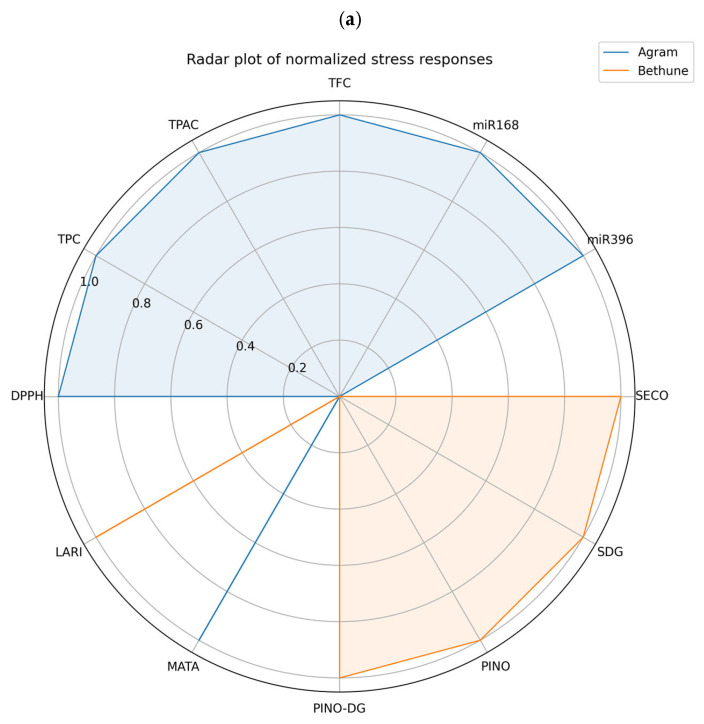

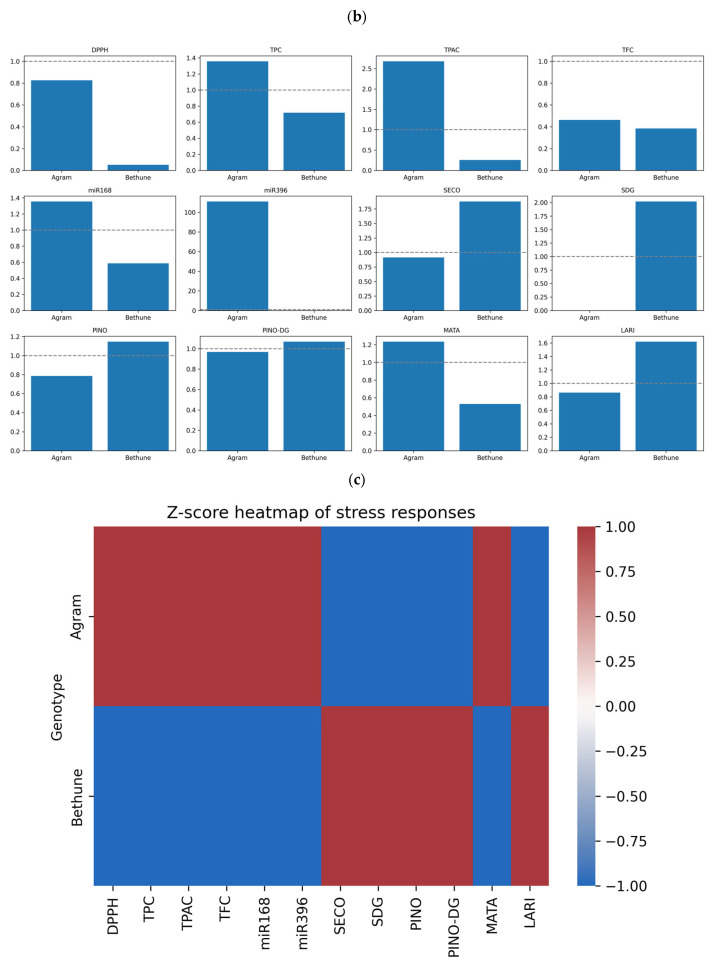
Integrated visualization of genotype-specific biochemical and molecular stress responses under salinity stress. (**a**) Radar plot showing normalized values of eleven biochemical and molecular stress-response markers in Agram (blue) and CDC Bethune (orange), highlighting contrasting response profiles across antioxidant, microRNA, and lignan-related traits. (**b**) Stress index analysis presented as bar diagrams, illustrating genotype- and trait-specific responses to salinity stress, including antioxidant activities, regulatory microRNAs, and lignan-associated metabolites. (**c**) Z-score-normalized heatmap depicting standardized stress responses of Agram and CDC Bethune across eleven biomarkers, with red and blue indicating positive and negative deviations from the mean, respectively. Hierarchical clustering reveals distinct metabolic response patterns between the two genotypes.

Radar plots require normalization to prevent any single variable from dominating the visualization; therefore, min–max normalization was applied. It converts all traits to the range of zero to one while preserving the relative differences. Each axis shows one trait. The distance from the center provides the normalized magnitude. Broad expansion of the radar shape denotes a strong multi-trait response, while a narrow shape indicates a weak or suppressed response; skewed shapes reflect selective pathway activation. The radar plot (Figure 5a) illustrates the normalized stress responses of Agram and CDC Bethune across twelve biochemical and molecular parameters, revealing a pronounced separation between the two genotypes, which occupy nearly opposite regions of the chart. Agram (blue) exhibited higher activity in the right and upper sectors of the radar plot, particularly for antioxidant- and microRNA-related putative markers, including DPPH, TFC, TPC, TPAC, miR168, and miR396. By contrast, CDC Bethune (orange) dominated the left and lower sectors, showing elevated levels of lignan-related metabolites such as SECO, SDG, PINO, PINO-DG, and LARI. For most variables, an inverse pattern of accumulation was observed between the two genotypes.

The bar diagrams provide the magnitude of each trait and the direction of change under stress. However, because bar plots are univariate, they do not reflect the integrated or overall stress response patterns. A stress index (SI) is computed here as the simple ratio of the value under NaCl and the value under control. The SI value of one indicates no change. Conversely, values greater than 1 indicate that the trait is induced under stress conditions. If SI < 1, the trait is observed to be suppressed. Where a value close to zero indicates a strong inhibition. The bar plot illustrates the magnitude of change, whereas the stress index reflects the strength of that change relative to the baseline. Stress index analysis using bar diagrams (Figure 5b) further indicated genotype- and trait-specific responses to salinity stress. Agram maintained higher levels of antioxidant-related traits, including TPAC and the two miRNAs. Conversely, CDC Bethune exhibited lower antioxidant activity and miRNA expression, alongside increased accumulation of lignan-related metabolites, particularly SECO, SDG, and LARI.

To enable cross-trait comparison, stress index values were standardized using Z-score normalization. For Z-score normalization, a multivariate matrix was generated in which genotypes and traits were defined as rows and columns, respectively. The Z-score is computed as shown below.

$$Z_{\mathrm{score}}=\frac{x-µ}{\Omega }$$

In this context, *x*, *μ*, and *Ω* correspond to the stress index of an individual genotype, the mean stress index across all genotypes, and the associated standard deviation, respectively. Using this Z-score, each trait is converted to a relative performance scale across genotypes. Z-scores less than zero represent below-average responses, whereas values greater than zero indicate above-average stress responses. Z-score-normalized heatmap analysis across the twelve biomarkers (Figure 5c) revealed two distinct metabolic clusters corresponding to the two genotypes. Agram displayed high positive Z-scores (approaching +1.0) for DPPH, TPC, TPAC, lus-miR168, and lus-miR396, and strongly negative Z-scores (approaching −1.0) for most lignan-related compounds. CDC Bethune showed the opposite pattern, with positive Z-scores for SECO, SDG, PINO, PINO_DG, and LARI, and negative scores for antioxidant and miRNA markers. Both genotypes exhibited positive Z-scores for MATA, although the magnitude was greater in Agram.

## 4. Discussion

Flax salt tolerance has become a focal point of research because salinity stress markedly impairs the productivity of this crop, which is otherwise valued for its high lignan biosynthetic potential and wide-ranging agronomic and industrial applications [33,34,35,36,37]. Lignans are recognized as important bioactive compounds that contribute to abiotic stress tolerance across diverse plant species, including *Sesamum indicum* L. [15], *Brassica napus* L. [16], and *Isatis indigotica* L. [38]. Nonetheless, relatively few studies have focused specifically on the involvement of lignans in salinity stress responses. The present study addresses this gap by comparing two flax genotypes with contrasting lignan contents under in vitro salinity stress conditions. An integrated metabolic, biochemical, and transcriptomic approach was employed to analyze salinity-induced responses, providing a comprehensive assessment of lignan-associated mechanisms contributing to salt stress mitigation in flax. Employing extreme genotypes is an established strategy in plant stress biology, especially for resolving and characterizing stress-associated responses [34,39,40]. The analysis of contrasting genotypes allows for more robust identification of stress-related differences and promotes hypothesis development concerning the contribution of selected metabolites to plant defense and adaptive responses.

The present study demonstrated that salinity stress significantly inhibited growth in both flax genotypes. Under controlled in vitro conditions, control plants completed normal growth within 15 days; in contrast, seeds exposed to 200 mM NaCl exhibited delayed germination and compromised development, resulting in failure of normal seedling establishment. The observed morphological changes were consistent with previously described and well-characterized salinity stress response mechanisms in plants [41] (Munns & Tester, 2008), suggesting that the applied stress level was biologically effective rather than purely detrimental. Salinity impedes seed germination by disrupting hormonal homeostasis, reducing gibberellic acid (GA) and increasing abscisic acid (ABA) levels, restricting water uptake, and suppressing α-amylase activity, resulting in pronounced delays even under moderate NaCl stress [42]. After 30 days, seedlings subjected to salinity stress were harvested; however, their growth remained markedly reduced compared with the control plants. Salt-treated seedlings exhibited morphological abnormalities, including thickened and withered leaves with yellowed tips and margins, as well as poorly developed root systems, compared to the well-formed shoots and roots of control plants. Comparable morphological abnormalities, including leaf thickening, chlorosis, and impaired root development, have been widely reported in various plant species exposed to salinity stress [43,44,45,46]. Notably, no significant differences in growth were detected between the two genotypes under either control or salinity-stress conditions, indicating that variation in lignan content did not influence overall plant growth in response to salinity.

Plants have evolved diverse adaptive mechanisms that enable varying levels of stress tolerance, largely determined by genetic plasticity and differences in morphological, molecular, biochemical, and metabolic pathways [5]. It is well established that, for most plants, the threshold level of soil salinity is approximately 40 mM NaCl [41]. However, experimental approaches aimed at investigating stress tolerance frequently apply higher salt concentrations to elicit measurable and reproducible responses under controlled conditions. Plants can perceive abiotic stresses and elicit appropriate responses with altered metabolism, growth and development [5]. Elicitation is one of the most effective biotechnological approaches used for the synthesis induction of specialized metabolites and stimulation of plant defense [47,48,49].

Previous studies on flax have employed a wide range of NaCl concentrations, typically between 50 mM and 250 mM, depending on the experimental objectives, exposure duration, developmental stage, and growth system used [25,33,34,39,40,50,51,52]. Importantly, the studies differ markedly in experimental design, including treatment duration—spanning short-term shock exposures to prolonged treatments of several weeks—as well as pre-treatment regimes that often involve seed germination or seedling establishment before stress imposition.

In our study, salinity stress was applied in vitro and immediately upon sowing, without a prior non-stress growth phase. This experimental setup differs from many previous reports but was intentionally chosen to expose seedlings to persistent salinity throughout early development. Compared to pot or soil systems, in vitro conditions generally require higher salt concentrations to induce comparable physiological and metabolic responses.

Drawing on a comprehensive literature survey and our prior pot-based experiments employing 100 mM NaCl [53], we determined that 200 mM NaCl represents an appropriate in vitro salinity treatment, providing robust differentiation between high- and low-lignan oil flax genotypes at molecular, biochemical, and metabolic levels. Comparable salinity levels have been successfully applied to discriminate between salt-sensitive and salt-tolerant flax cultivars, notably in large-scale genome-wide association studies of genetically diverse flax accessions [33,34]. Additionally, Wang et al. (2022) applied similar salinity levels, including 200 mM NaCl, to investigate macroscopic responses of both oilseed and fiber flax under acute salt stress [40].

A further consideration in selecting this concentration was our aim to explore the biological role of lignans in defense or adaptation mechanisms under severe stress conditions, rather than to simulate agronomic performance. While we acknowledge that such salinity levels would likely be detrimental under field conditions, the applied stress allowed us to capture metabolically meaningful responses in lignan biosynthesis.

The applied NaCl concentration was deliberately chosen to induce a strong stress response over the extended cultivation period (30 days), during which both the early osmotic phase—characterized by growth inhibition—and the later ionic phase—associated with salt accumulation in tissues—are expected to occur [41]. Within this framework, the observed molecular, biochemical, and metabolic changes were interpreted as indicators of overall defense and adaptation capacity mediated by lignans, rather than as temporally resolved stress responses. We recognize that an experimental design in which stress and control samples are evaluated at the same developmental stage would provide a clearer separation of these factors.

In the present study, however, we intentionally adopted a continuous stress exposure model, in which flax seeds were subjected to salinity immediately upon sowing, without a prior non-stress growth phase. This choice was motivated by a biological rather than a purely statistical consideration, as salt stress studies in plants differ widely in methodology, including seed pre-germination, delayed stress application, and highly variable treatment durations varying from short-term shock exposures to long-term cultivation periods [25,33,34,39,40,52]. Our objective was to assess plant responses to persistent, cumulative stress rather than stress applied at a defined developmental checkpoint.

Plant lignans are substances of immense importance and potential, both for the plant organism itself and human nutrition and health. Lignans have been identified in various plant organs, including flowers, seeds, leaves, roots, fruits, and woody tissues. The key feature of their role in plants is their antioxidant activity during defense responses [15]. Their accumulation is enhanced under abiotic stress conditions such as drought, salinity, extreme temperatures, heavy-metal contamination, and UV radiation [13,14,15,54,55,56].

Due to their phenolic structure, lignans act as hydroxyl-radical scavengers, protecting the organism from oxidative damage to lipids, proteins, nucleic acids, and tissues [57]. Flax seeds are one of the principal sources of lignans, with SECO occurring predominantly in its glycosylated form as SDG, the most abundant lignan, while other lignans such as MATA, PINO, and LARI, though present in smaller amounts, also contribute significant value [58].

Although the present study builds conceptually on our earlier work published [53], that study was designed as a methodologically comprehensive pilot investigation, focusing on lignan-related miRNAs, antioxidant enzyme activities, and detailed morphological traits under controlled conditions. In contrast, the present manuscript was designed with a focused, metabolite-centered objective, explicitly targeting the characterization of stress-induced alterations in individual lignan compounds under high-salinity conditions. We examined the effect of salt stress conditions on lignan composition and content and quantified selected lignans using HPLC-UV-MS/MS analysis. NaCl stress resulted in a significant reduction of PINO, SDG and LARI in Agram. In CDC Bethune, exposure to NaCl resulted in an approximately 2-fold or 1.5–2-fold increase in the contents of SDG, LARI, SECO, and PINO, respectively, compared with the control. In both genotypes, PINO was produced at levels several times higher than those of the other lignans. Notably, three previously uncharacterized PINO derivatives were detected in both genotypes at concentrations exceeding those of PINO and PDG. These derivatives exhibited retention times between PDG and PINO and showed identical MRM transitions to PINO, indicating structural similarity. The accumulation patterns of the PINO derivatives differed between genotypes and treatments. PINO derivative 1 was reduced by more than tenfold in NaCl-stressed CDC Bethune plants compared with the respective control, whereas in Agram the reduction was limited to approximately 50% of control levels. A similar genotype-dependent response was observed for PINO derivative 2. In contrast, PINO derivative 3 showed no apparent dependence on genotype or NaCl treatment. De novo biosynthesis of flax lignans in response to pathogen infection has been documented in several studies [59,60]. However, future studies involving a substantially higher number of genotypes are necessary to assess the consistency and predictive value of these features for the feasibility of multi-level profiling as a selection tool.

Flaxseed contains a wide array of bioactive metabolites, and its overall biological potential arises from the complex interactions among metabolic pathways and regulatory networks operating at genetic and epigenetic levels. Accordingly, plant defense and adaptation to abiotic stress represent integrated processes rather than the action of a single compound class. The content of lignans in plant tissues is affected by genomic, physiological, biochemical, and environmental factors. However, genotype is the major factor affecting the lignan content [54,55]. Previous research has demonstrated pronounced genotype- dependent variation in flaxseed composition, including differences in mineral content, phenolic acid profiles, fiber composition, and antioxidant and antimicrobial activities [61].

Salinity stress is widely recognized as a key modulator of plant secondary metabolite production under controlled in vitro conditions [62]. Phenolic compounds, including flavonoids, phenolic acids, and other polyphenols, exhibit strong redox properties that confer antioxidant capacity. These metabolites frequently accumulate in response to environmental stress and are therefore considered reliable biochemical putative markers of plant stress responses [63]. In line with their established role in scavenging stress-induced reactive oxygen species (ROS), enhanced accumulation of phenolic compounds has been reported under moderate to severe salinity stress [14,64]. Although NaCl treatment commonly stimulates total flavonoid content (TFC), exposure to excessively high salinity levels (e.g., 200 mM NaCl) has been shown to suppress TFC in several plant species [65,66,67]. In the present study, salt stress resulted in a marked reduction in TFC in both genotypes, indicating that flavonoid biosynthesis is particularly sensitive to elevated salinity. No significant differences in TFC were observed between the two genotypes under salt treatment. A genotype-specific response was observed for total phenolic content (TPC) and total phenolic acid content (TPAC). In CDC Bethune, both TPC and TPAC declined under salt stress, mirroring the trend observed for TFC. In contrast, Agram exhibited an increase in both TPC and TPAC even at high NaCl concentrations. Consequently, unlike TFC, both TPC and TPAC were higher in Agram than in CDC Bethune under salinity stress. Abiotic stress conditions are known to activate the phenylpropanoid biosynthetic pathway, leading to the enhanced synthesis of diverse phenolic metabolites [14]. However, changes in TFC, TPC, and TPAC vary in both magnitude and direction depending on salinity intensity, genotype, and experimental condition [68,69,70,71].

In CDC Bethune, salinity stress was associated with an increased synthesis of specific lignans, suggesting activation of lignan-related metabolic pathways under stress conditions. However, this metabolic response was not accompanied by an increase in total antioxidant activity or antioxidant enzyme responses. We interpret this pattern as an indication that lignan accumulation and antioxidant capacity may represent distinct, only partially overlapping defense strategies, rather than directly coupled processes. Importantly, lignans are not necessarily expected to function as primary or immediate antioxidants. While some lignan compounds may exhibit antioxidant properties, their biological role in plants is often considered broader. In this context, the increased lignan accumulation observed in CDC Bethune may reflect a metabolic or structural adaptive response, whereas the lower antioxidant activity could indicate either reduced reliance on enzymatic ROS scavenging or differences in stress perception and ROS generation. Therefore, the observed relationship does not imply that higher lignan levels should necessarily correlate with higher antioxidant activity. Instead, our results suggest that CDC Bethune may employ a distinct adaptive strategy, characterized by enhanced lignan biosynthesis under salinity stress while maintaining comparatively lower antioxidant responses.

The data clearly indicates that salinity stress defense responses are strongly influenced by genotype. As noted, the Agram genotype appears to preferentially activate defense mechanisms associated with enhanced antioxidant capacity, largely mediated by polyphenolic compounds, together with distinct patterns of miRNA regulation. In contrast, CDC Bethune responds to salinity stress predominantly through the induction of lignan biosynthesis, reflected by increased accumulation of specific lignan compounds. It is important to emphasize that, given the severity of the applied NaCl concentration, none of the observed defense responses were sufficient to prevent eventual plant death. Thus, the objective of the study was not to evaluate agronomic tolerance, but to dissect and compare stress-responsive defense strategies acting within a single species under controlled, high-intensity stress conditions.

MicroRNAs (miRNAs) are endogenous 19–24 bp long molecules of non-coding single-stranded RNA that regulate gene expression by inhibiting gene translation or promoting cleavage of target post-transcriptional mRNA [72]. Plant miRNAs are involved in responses to abiotic stresses of various natures, including low temperature, drought, salinity, oxidative stress, UV-B radiation, heavy metals, as well as biotic stresses, hence acting as stress-sensitive markers [73,74,75,76,77,78]. They are particularly important in plant growth and development, hormone regulation, organ differentiation, alternative splicing and metabolite accumulation [79,80,81]. Several miRNAs regulate the lignan biosynthetic pathway [11].

The expression levels of two lignan biosynthesis-related miRNAs, miR168a and miR396, were analyzed using the TT-qPCR assay. This assay was originally developed by Androvic et al. [31] for miRNA profiling in mouse tissues and has since been successfully applied to quantify plant miRNA expression [53,82,83,84,85]. A previous study reported the involvement of miR168 and miR396 in the regulation of cytochrome P450 monooxygenase CYP71E, while miR396 is also involved in the regulation of secoisolariciresinol dehydrogenase (SDH) [86]. CYP71E is found to be involved in cyanogenic glycosides biosynthesis [87] while SDH converts the SECO to MATA [88]. In the present study, miR168 exhibited contrasting expression patterns under salt stress, being upregulated in Agram and downregulated in CDC Bethune relative to their respective controls. Despite this differential regulation, the overall expression level of miR168 was higher in CDC Bethune than in Agram. Similarly, miR396 was upregulated in Agram but downregulated in CDC Bethune under salt stress; however, in contrast to miR168, the expression level of miR396 was higher in Agram than in CDC Bethune. The contrasting regulation of both miRNAs under salt stress between the two genotypes may support our hypothesis that miRNAs could play a role in plant stress responses, possibly contributing to the regulation of lignan biosynthesis.

One of the target sequences of the miR168 family is the cytochrome P450 sequence. This sequence is involved in a wide range of biosynthetic reactions, including the biosynthesis of stress-related fatty acids [89]. The miR168 microRNA controls its own expression by binding to specific proteins involved in the post-transcriptional gene silencing pathway. It also regulates the function of all microRNAs whose regulatory target is AGO1 expression [90,91]. The miR168 microRNA is also considered a potential biomarker of abiotic stress [89].

MADS-box transcription factors play critical roles in plant development and stress responses. The study [92] showed that lus-miR396 and lus-miR156 are the main miRNAs targeting the LuMADS gene family. Growth regulatory factor (GRF) proteins constitute a plant-specific family of transcription factors involved in the regulation of diverse processes related to plant growth and development. Bioinformatics analysis of the GRF family in flax predicted 17 LuGRF genes, out of which 15 were predicted to be regulated by lus-miR396 miRNA [93]. MYB transcription factors are known regulators of flavonoid biosynthesis. It has been discovered that miR396 negatively regulates flavonoid biosynthesis by repressing GbMYB41 expression. Consequently, the miR396–GbMYB41 module was identified as a key regulatory component involved in flavonoid biosynthesis [94]. A crucial regulatory module miR396-GRF/GIF—consisting of microRNA miR396, GROWTH REGULATING FACTORS (GRFs) and GRF-INTERACTING FACTORS (GIFs)—affects agronomically important traits, including its role in coordinating growth with endogenous and environmental factors [95].

The contrasting spatial distributions observed in the radar plot indicate that Agram and CDC Bethune employ fundamentally different strategies to cope with salinity-induced stress. The predominance of antioxidant- and miRNA-associated putative markers in Agram suggests a stress response primarily driven by redox regulation and post-transcriptional control mechanisms. The strong induction of TPAC and regulatory miRNAs such as miR168 and miR396 points toward an adaptive regulatory strategy that may facilitate growth modulation and enhanced control of ROS under stress conditions. In contrast, the enrichment of lignan-related metabolites in CDC Bethune, coupled with suppressed antioxidant activity and miRNA expression, suggests a stress-response strategy centered on the phenylpropanoid pathway. The near mutual exclusivity of many putative biomarkers between the two genotypes, as evident from both radar plot and Z-score heatmap analyses, indicates that these differences reflect divergent molecular stress-response pathways rather than simple variation in stress sensitivity. The shared induction of MATA in both genotypes suggests the presence of a conserved component of the stress response, although its stronger expression in Agram may reflect differential pathway integration.

## 5. Conclusions

The present study focused specifically on lignans and their potential involvement in stress-responsive defense mechanisms, particularly in relation to miRNA-mediated regulation. Both genotypes possess the genetic capacity for lignan biosynthesis; however, they differ markedly in total lignan content as well as in the relative abundance of individual lignan compounds. These observations suggest that the lignan biosynthetic machinery is largely conserved in flax but is differentially expressed or regulated in a genotype-specific manner. Based on this, we propose that it may be of interest in future studies to test whether lignan biosynthetic pathways can be stimulated or upregulated in genotypes or plant species that naturally accumulate only low levels of these compounds. While the results provide insight into genotype-specific stress response strategies, further validation across multiple genotypes, stress intensities, and growth conditions would be required before any application to selection or breeding contexts can be considered.

## Figures and Tables

**Table 1:** Lignan contents (µg/g, fresh weight) in control plants and stressed plants with 200 mM NaCl.

Genotypes	SECO	SDG	PINO	PINO-DG	LARI	MATA
Agram control	6.14	0.09	23.23	3.46	7.57	0.09
Agram NaCl	5.6	0.18	18.24	3.35	6.53	0.12
CDC Bethune control	3.57	0.39	10.27	3.82	3.06	0.09
CDC Bethune NaCl	6.71	0.78	11.77	4.08	4.95	0.05

SECO—secoisolariciresinol; SDG—Secoisolariciresinol diglucoside; LARI—Lariciresinol; MATA—Matariresinol; PINO—Pinoresinol; PINO-DG—Pinoresinol diclucoside.

## Data Availability

The datasets presented in this article are not readily available because the data are part of an ongoing research project. Requests to access the datasets should be directed to the corresponding author (K.R.).

## Abbreviations

The following abbreviations are used in this manuscript:

CAE	Caffeic acid equivalents
DPPH	2,2-diphenyl-1-picrylhydrazyl
GAE	Gallic acid equivalents
HPLC	High-Performance Liquid Chromatography
LARI	Lariciresinol
MATA	Matairesinol
miRNAs	MicroRNAs
MS	Mass Spectrometry
PINO	Pinoresinol
PINO-DG	Pinoresinol diglucoside
QE	Quercetin equivalents
ROS	Reactive oxygen species
SDG	Secoisolariciresinol diglucoside
SECO	Secoisolariciresinol
TEAC	Trolox equivalent antioxidant capacity
TFC	Total flavonoid content
TPC	Total polyphenol content
TPAC	Total phenolic acid content

## References

[1] FAO (2024). FAO Launches First Major Global Assessment of Salt-Affected Soils in 50 Years.

[2] Zhao S., Zhang Q., Liu M., Zhou H., Ma C., Wang P. (2021). Regulation of Plant Responses to Salt Stress. Int. J. Mol. Sci..

[3] Ma Y., Dias M.C., Freitas H. (2020). Drought and Salinity Stress Responses and Microbe-Induced Tolerance in Plants. Front. Plant Sci..

[4] Türkan I., Demiral T. (2009). Recent Developments in Understanding Salinity Tolerance. Environ. Exp. Bot..

[5] Bartels D., Sunkar R. (2005). Drought and Salt Tolerance in Plants. Crit. Rev. Plant Sci..

[6] Hasanuzzaman M., Bhuyan M.H.M.B., Anee T.I., Parvin K., Nahar K., Mahmud J.A., Fujita M. (2019). Regulation of Ascorbate-Glutathione Pathway in Mitigating Oxidative Damage in Plants under Abiotic Stress. Antioxidants.

[7] Mekawy A.M.M., Assaha D.V.M., Ueda A. (2020). Differential Salt Sensitivity of Two Flax Cultivars Coincides with Differential Sodium Accumulation, Biosynthesis of Osmolytes and Antioxidant Enzyme Activities. J. Plant Growth Regul..

[8] Gill S.S., Tuteja N. (2010). Reactive Oxygen Species and Antioxidant Machinery in Abiotic Stress Tolerance in Crop Plants. Plant Physiol. Biochem..

[9] Šamec D., Karalija E., Šola I., Vujčić Bok V., Salopek-Sondi B. (2021). The Role of Polyphenols in Abiotic Stress Response: The Influence of Molecular Structure. Plants.

[10] Teponno R.B., Kusari S., Spiteller M. (2016). Recent Advances in Research on Lignans and Neolignans. Nat. Prod. Rep..

[11] Ražná K., Nôžková J., Vargaová A., Harenčár Ľ., Bjelková M. (2022). Biological Functions of Lignans in Plants. Agriculture.

[12] Markulin L., Drouet S., Corbin C., Decourtil C., Garros L., Renouard S., Lopez T., Mongelard G., Gutierrez L., Auguin D. (2019). The Control Exerted by ABA on Lignan Biosynthesis in Flax (*Linum usitatissimum* L.) Is Modulated by a Ca^2+^ Signal Transduction Involving the Calmodulin-like LuCML15b. J. Plant Physiol..

[13] Markulin L., Corbin C., Renouard S., Drouet S., Gutierrez L., Mateljak I., Auguin D., Hano C., Fuss E., Lainé E. (2019). Pinoresinol–Lariciresinol Reductases, Key to the Lignan Synthesis in Plants. Planta.

[14] Sharma A., Shahzad B., Rehman A., Bhardwaj R., Landi M., Zheng B. (2019). Response of Phenylpropanoid Pathway and the Role of Polyphenols in Plants under Abiotic Stress. Molecules.

[15] Ghotbzadeh Kermani S., Saeidi G., Sabzalian M.R., Gianinetti A. (2019). Drought Stress Influenced Sesamin and Sesamolin Content and Polyphenolic Components in Sesame (*Sesamum indicum* L.) Populations with Contrasting Seed Coat Colors. Food Chem..

[16] Mwamba T.M., Islam F., Ali B., Lwalaba J.L.W., Gill R.A., Zhang F., Farooq M.A., Ali S., Ulhassan Z., Huang Q. (2020). Comparative Metabolomic Responses of Low- and High-Cadmium Accumulating Genotypes Reveal the Cadmium Adaptive Mechanism in *Brassica napus*. Chemosphere.

[17] Zálešák F., Bon D.J.-Y.D., Pospíšil J. (2019). Lignans and Neolignans: Plant Secondary Metabolites as a Reservoir of Biologically Active Substances. Pharmacol. Res..

[18] Li Z., Xu R., Li N. (2018). MicroRNAs from Plants to Animals, Do They Define a New Messenger for Communication?. Nutr. Metab..

[19] Reinhart B.J., Weinstein E.G., Rhoades M.W., Bartel B., Bartel D.P. (2002). MicroRNAs in Plants. Genes Dev..

[20] Islam W., Waheed A., Naveed H., Zeng F. (2022). MicroRNAs Mediated Plant Responses to Salt Stress. Cells.

[21] Baek D., Chun H.J., Kang S., Shin G., Park S.J., Hong H., Kim C., Kim D.H., Lee S.Y., Kim M.C. (2016). A Role for Arabidopsis *miR399f* in Salt, Drought, and ABA Signaling. Mol. Cells.

[22] Cheng X., He Q., Tang S., Wang H., Zhang X., Lv M., Liu H., Gao Q., Zhou Y., Wang Q. (2021). The miR172/IDS1 Signaling Module Confers Salt Tolerance through Maintaining ROS Homeostasis in Cereal Crops. New Phytol..

[23] Goyal A., Sharma V., Upadhyay N., Gill S., Sihag M. (2014). Flax and Flaxseed Oil: An Ancient Medicine & Modern Functional Food. J. Food Sci. Technol..

[24] Rodríguez-García C., Sánchez-Quesada C., Toledo E., Delgado-Rodríguez M., Gaforio J.J. (2019). Naturally Lignan-Rich Foods: A Dietary Tool for Health Promotion?. Molecules.

[25] Yadav N., Sawariya M., Kumar A., Mehra H., Sharma J., Kumar S., Devi S., Kaur V., Arya S.S. (2024). Influence of GA3 (Gibberellic Acid) and Ca(Calcium) on Root Trait Variation and Osmotic Potential of Linseed (*Linum usitatissimum* L.) under Chloride-Dominated Salinity. J. Appl. Nat. Sci..

[26] Popova I.E., Hall C., Kubátová A. (2009). Determination of Lignans in Flaxseed Using Liquid Chromatography with Time-of-Flight Mass Spectrometry. J. Chromatogr. A.

[27] Sánchez-Moreno C., Larrauri J.A., Saura-Calixto F. (1998). A Procedure to Measure the Antiradical Efficiency of Polyphenols. J. Sci. Food Agric..

[28] Singleton V.L., Rossi J.A. (1965). Colorimetry of Total Phenolics with Phosphomolybdic-Phosphotungstic Acid Reagents. Am. J. Enol. Vitic..

[29] Jain R., Rao B., Tare A.B. (2017). Comparative Analysis of the Spectrophotometry Based Total Phenolic Acid Estimation Methods. J. Anal. Chem..

[30] Willett W.C. (2002). Balancing Life-Style and Genomics Research for Disease Prevention. Science.

[31] Androvic P., Valihrach L., Elling J., Sjoback R., Kubista M. (2017). Two-Tailed RT-qPCR: A Novel Method for Highly Accurate miRNA Quantification. Nucleic Acids Res..

[32] Paolacci A.R., Tanzarella O.A., Porceddu E., Ciaffi M. (2009). Identification and Validation of Reference Genes for Quantitative RT-PCR Normalization in Wheat. BMC Mol. Biol..

[33] Li X., Guo D., Xue M., Li G., Yan Q., Jiang H., Liu H., Chen J., Gao Y., Duan L. (2022). Genome-Wide Association Study of Salt Tolerance at the Seed Germination Stage in Flax (*Linum usitatissimum* L.). Genes.

[34] Li Y., Chen J., Li X., Jiang H., Guo D., Xie F., Zhang Z., Xie L. (2022). Adaptive Response and Transcriptomic Analysis of Flax (*Linum usitatissimum* L.) Seedlings to Salt Stress. Genes.

[35] Li Y.-D., Ma R., Zheng Y.-X., Xie L.-Q. (2025). Genome-Wide Identification of APX Genes in Flax (*Linum usitatissimum*) and Functional Characterization of *LuAPX12* in Osmotic and Salinity Stress Responses. BMC Plant Biol..

[36] Hussein H.-A.A. (2024). Thiourea Induces Antioxidant Mechanisms of Salt Tolerance in Flax Plants. Physiol. Mol. Biol. Plants.

[37] Zhang Y., Wang R., Wang H., Wang H. (2025). *LuCSD3* Enhances Salt Stress Tolerance in Flax: Genome-Wide Profiling and Functional Validation of the SOD Gene Family. Front. Plant Sci..

[38] Xiao Y., Feng J., Li Q., Zhou Y., Bu Q., Zhou J., Tan H., Yang Y., Zhang L., Chen W. (2020). *Ii*WRKY34 Positively Regulates Yield, Lignan Biosynthesis and Stress Tolerance in *Isatis indigotica*. Acta Pharm. Sin. B.

[39] Kawasaki S., Borchert C., Deyholos M., Wang H., Brazille S., Kawai K., Galbraith D., Bohnert H.J. (2001). Gene Expression Profiles during the Initial Phase of Salt Stress in Rice. Plant Cell.

[40] Wang N., Lin Y., Qi F., Xiaoyang C., Peng Z., Yu Y., Liu Y., Zhang J., Qi X., Deyholos M. (2022). Comprehensive Analysis of Differentially Expressed Genes and Epigenetic Modification-Related Expression Variation Induced by Saline Stress at Seedling Stage in Fiber and Oil Flax, *Linum usitatissimum* L.. Plants.

[41] Munns R., Tester M. (2008). Mechanisms of Salinity Tolerance. Annu. Rev. Plant Biol..

[42] Atta K., Mondal S., Gorai S., Singh A.P., Kumari A., Ghosh T., Roy A., Hembram S., Gaikwad D.J., Mondal S. (2023). Impacts of Salinity Stress on Crop Plants: Improving Salt Tolerance through Genetic and Molecular Dissection. Front. Plant Sci..

[43] Taïbi K., Taïbi F., Ait Abderrahim L., Ennajah A., Belkhodja M., Mulet J.M. (2016). Effect of Salt Stress on Growth, Chlorophyll Content, Lipid Peroxidation and Antioxidant Defence Systems in *Phaseolus vulgaris* L.. S. Afr. J. Bot..

[44] Ji X., Tang J., Zhang J. (2022). Effects of Salt Stress on the Morphology, Growth and Physiological Parameters of *Juglans microcarpa* L. Seedlings. Plants.

[45] Lu C., Li L., Liu X., Chen M., Wan S., Li G. (2023). Salt Stress Inhibits Photosynthesis and Destroys Chloroplast Structure by Downregulating Chloroplast Development–Related Genes in *Robinia pseudoacacia* Seedlings. Plants.

[46] Liu H., Ding D., Sun Y., Ma R., Yang X., Liu J., Zhang G. (2025). Salt Stress Leads to Morphological and Transcriptional Changes in Roots of Pumpkins (*Cucurbita* spp.). Plants.

[47] Hano C., Martin I., Fliniaux O., Legrand B., Gutierrez L., Arroo R.R.J., Mesnard F., Lamblin F., Lainé E. (2006). Pinoresinol-Lariciresinol Reductase Gene Expression and Secoisolariciresinol Diglucoside Accumulation in Developing Flax (*Linum usitatissimum*) Seeds. Planta.

[48] Ramirez-Estrada K., Vidal-Limon H., Hidalgo D., Moyano E., Golenioswki M., Cusidó R.M., Palazon J. (2016). Elicitation, an Effective Strategy for the Biotechnological Production of Bioactive High-Added Value Compounds in Plant Cell Factories. Molecules.

[49] Khan I., Khan M.A., Shehzad M.A., Ali A., Mohammad S., Ali H., Alyemeni M.N., Ahmad P. (2020). Micropropagation and Production of Health Promoting Lignans in *Linum usitatissimum*. Plants.

[50] Wu J., Zhao Q., Wu G., Yuan H., Ma Y., Lin H., Pan L., Li S., Sun D. (2019). Comprehensive Analysis of Differentially Expressed Unigenes under NaCl Stress in Flax (*Linum usitatissimum* L.) Using RNA-Seq. Int. J. Mol. Sci..

[51] Hussein H.-A.A., Alshammari S.O. (2022). Cysteine Mitigates the Effect of NaCl Salt Toxicity in Flax (*Linum usitatissimum* L) Plants by Modulating Antioxidant Systems. Sci. Rep..

[52] Robin A.H.K., Matthew C., Uddin M.J., Bayazid K.N. (2016). Salinity-Induced Reduction in Root Surface Area and Changes in Major Root and Shoot Traits at the Phytomer Level in Wheat. J. Exp. Bot..

[53] Debnath A.J., Harenčár Ľ., Kučka M., Kovár M., Ivanišová E., Mistríková V., Gažo J., Ražná K. (2025). A Comparative Analysis of the Adaptability of Salt Stress between Two Flax (*Linum usitatissimum* L.) Genotypes, Flanders and Astella, Having Contrasting Lignan Contents. Planta.

[54] Garros L., Drouet S., Corbin C., Decourtil C., Fidel T., Lebas de Lacour J., Leclerc E.A., Renouard S., Tungmunnithum D., Doussot J. (2018). Insight into the Influence of Cultivar Type, Cultivation Year, and Site on the Lignans and Related Phenolic Profiles, and the Health-Promoting Antioxidant Potential of Flax (*Linum usitatissimum* L.) Seeds. Molecules.

[55] Kyselka J., Rabiej D., Dragoun M., Kreps F., Burčová Z., Němečková I., Smolová J., Bjelková M., Szydłowska-Czerniak A., Schmidt Š. (2017). Antioxidant and Antimicrobial Activity of Linseed Lignans and Phenolic Acids. Eur. Food Res. Technol..

[56] Heldt H.-W., Piechulla B. (2021). Plant Biochemistry.

[57] Kajla P., Sharma A., Sood D.R. (2015). Flaxseed—A Potential Functional Food Source. J. Food Sci. Technol..

[58] Sangiorgio P., Errico S., Verardi A., Moliterni S., Tamasi G., Rossi C., Balducchi R. (2023). Bioactive Lignans from Flaxseed: Biological Properties and Patented Recovery Technologies. Nutraceuticals.

[59] Zeitoun A.M., Preisner M., Kulma A., Dymińska L., Hanuza J., Starzycki M., Szopa J. (2014). Does Biopolymers Composition in Seeds Contribute to the Flax Resistance against the Fusarium Infection?. Biotechnol. Prog..

[60] Oros G., Kállai Z., Jogaiah S., Abdelrahman M. (2019). Phytoanticipins: The Constitutive Defense Compounds as Potential Botanical Fungicides. Bioactive Molecules in Plant Defense: Signaling in Growth and Stress.

[61] Kučka M., Harenčár Ľ., Ražná K., Nôžková J., Kowalczewski P.Ł., Deyholos M., Dziedzic K., Rybicka I., Zembrzuska J., Kačániová M. (2024). Great Potential of Flaxseed Mucilage. Eur. Food Res. Technol..

[62] Razzaq A., Zafar M.M., Ali A., Ihsan L., Qadir F., Khan M.N., Zhang Y., Gao L., Cong H., Iqbal R. (2025). Elicitor-Mediated Enhancement of Secondary Metabolites in Plant Species: A Review. Front. Plant Sci..

[63] Humbal A., Pathak B. (2023). Influence of Exogenous Elicitors on the Production of Secondary Metabolite in Plants: A Review (“VSI: Secondary Metabolites”). Plant Stress.

[64] Rodrigues M.J., Neng N., Custódio L. (2024). NaCl Elicitation Enhances Metabolite Accumulation and Stress Resilience in *Inula crithmoides* L. Shoot Cultures: Implications for Its Nutritional and Medicinal Value. Plant Cell Tissue Organ Cult..

[65] Hand M.J., Taffouo V.D., Nouck A.E., Nyemene K.P.J., Tonfack B., Meguekam T.L., Youmbi E. (2017). Effects of Salt Stress on Plant Growth, Nutrient Partitioning, Chlorophyll Content, Leaf Relative Water Content, Accumulation of Osmolytes and Antioxidant Compounds in Pepper (*Capsicum annuum* L.) Cultivars. Not. Bot. Horti Agrobot. Cluj-Napoca.

[66] Pungin A., Lartseva L., Loskutnikova V., Shakhov V., Popova E., Skrypnik L., Krol O. (2023). Effect of Salinity Stress on Phenolic Compounds and Antioxidant Activity in Halophytes *Spergularia marina* (L.) Griseb. and *Glaux maritima* L. Cultured In Vitro. Plants.

[67] Mohammad S.M., Khalid Y.A., Sawsan A.O. (2025). Impact of Salinity Stress on Phytochemical Profiles in Salvia Officinalis. Plant Sci. Today.

[68] Slama I., M’Rabet R., Ksouri R., Talbi O., Debez A., Abdelly C. (2017). Effects of Salt Treatment on Growth, Lipid Membrane Peroxidation, Polyphenol Content, and Antioxidant Activities in Leaves of *Sesuvium portulacastrum* L.. Arid. Land Res. Manag..

[69] Linić I., Šamec D., Grúz J., Vujčić Bok V., Strnad M., Salopek-Sondi B. (2019). Involvement of Phenolic Acids in Short-Term Adaptation to Salinity Stress Is Species-Specific among Brassicaceae. Plants.

[70] Sarker U., Hossain M.N., Oba S., Ercisli S., Marc R.A., Golokhvast K.S. (2023). Salinity Stress Ameliorates Pigments, Minerals, Polyphenolic Profiles, and Antiradical Capacity in Lalshak. Antioxidants.

[71] Azeem M., Pirjan K., Qasim M., Mahmood A., Javed T., Muhammad H., Yang S., Dong R., Ali B., Rahimi M. (2023). Salinity Stress Improves Antioxidant Potential by Modulating Physio-Biochemical Responses in *Moringa oleifera* Lam. Sci. Rep..

[72] Zhou R., Yu X., Ottosen C.-O., Zhang T., Wu Z., Zhao T. (2020). Unique miRNAs and Their Targets in Tomato Leaf Responding to Combined Drought and Heat Stress. BMC Plant Biol..

[73] Yu Y., Wu G., Yuan H., Cheng L., Zhao D., Huang W., Zhang S., Zhang L., Chen H., Zhang J. (2016). Identification and Characterization of miRNAs and Targets in Flax (*Linum usitatissimum*) under Saline, Alkaline, and Saline-Alkaline Stresses. BMC Plant Biol..

[74] Li S., Castillo-González C., Yu B., Zhang X. (2017). The Functions of Plant Small RNAs in Development and in Stress Responses. Plant J..

[75] Roy Chowdhury M., Basak J. (2019). Tiny Yet Indispensable Plant MicroRNAs Are Worth to Explore as Key Components for Combating Genotoxic Stresses. Front. Plant Sci..

[76] Moumita R.C., Jolly B., Ranjit P.B. (2020). Elucidating the Functional Role of Predicted miRNAs in Post-Transcriptional Gene Regulation Along with Symbiosis in *Medicago truncatula*. Curr. Bioinform..

[77] Millar A.A. (2020). The Function of miRNAs in Plants. Plants.

[78] Dong Q., Hu B., Zhang C. (2022). microRNAs and Their Roles in Plant Development. Front. Plant Sci..

[79] Gupta O.P., Karkute S.G., Banerjee S., Meena N.L., Dahuja A. (2017). Contemporary Understanding of miRNA-Based Regulation of Secondary Metabolites Biosynthesis in Plants. Front. Plant Sci..

[80] Xu X., Chen X., Chen Y., Zhang Q., Su L., Chen X., Chen Y., Zhang Z., Lin Y., Lai Z. (2020). Genome-Wide Identification of miRNAs and Their Targets during Early Somatic Embryogenesis in *Dimocarpus longan* Lour. Sci. Rep..

[81] Arazi T., Khedia J. (2022). Tomato MicroRNAs and Their Functions. Int. J. Mol. Sci..

[82] Anna B.-B., Grzegorz B., Marek K., Piotr G., Marcin F. (2019). Exposure to High-Intensity Light Systemically Induces Micro-Transcriptomic Changes in Arabidopsis Thaliana Roots. Int. J. Mol. Sci..

[83] Pawełkowicz M.E., Skarzyńska A., Koter M.D., Turek S., Pląder W. (2022). miRNA Profiling and Its Role in Multi-Omics Regulatory Networks Connected with Somaclonal Variation in Cucumber (*Cucumis sativus* L.). Int. J. Mol. Sci..

[84] Barczak-Brzyżek A., Brzyżek G., Koter M., Siedlecka E., Gawroński P., Filipecki M. (2022). Plastid Retrograde Regulation of miRNA Expression in Response to Light Stress. BMC Plant Biol..

[85] Kong L., Zhuo Y., Xu J., Meng X., Wang Y., Zhao W., Lai H., Chen J., Wang J. (2022). Identification of Long Non-Coding RNAs and microRNAs Involved in Anther Development in the Tropical Camellia Oleifera. BMC Genom..

[86] Harenčár Ľ., Ražná K. (2024). In Silico Prediction of microRNA Families Involved in the Biosynthesis of Lignans and Cyanogenic Glycosides in Flax (*Linum usitatissimum* L.). Plant Growth Regul..

[87] An F., Xiao X., Chen T., Xue J., Luo X., Ou W., Li K., Cai J., Chen S. (2022). Systematic Analysis of bHLH Transcription Factors in Cassava Uncovers Their Roles in Postharvest Physiological Deterioration and Cyanogenic Glycosides Biosynthesis. Front. Plant Sci..

[88] Kezimana P., Dmitriev A.A., Kudryavtseva A.V., Romanova E.V., Melnikova N.V. (2018). Secoisolariciresinol Diglucoside of Flaxseed and Its Metabolites: Biosynthesis and Potential for Nutraceuticals. Front. Genet..

[89] Bej S., Basak J. (2014). MicroRNAs: The Potential Biomarkers in Plant Stress Response. Am. J. Plant Sci..

[90] Gazzani S., Li M., Maistri S., Scarponi E., Graziola M., Barbaro E., Wunder J., Furini A., Saedler H., Varotto C. (2009). Evolution of MIR168 Paralogs in Brassicaceae. BMC Evol. Biol..

[91] Li W., Cui X., Meng Z., Huang X., Xie Q., Wu H., Jin H., Zhang D., Liang W. (2012). Transcriptional Regulation of Arabidopsis MIR168a and Argonaute1 Homeostasis in Abscisic Acid and Abiotic Stress Responses. Plant Physiol..

[92] Lu J., Wu H., Pitt D.M., Liu X., Song X., Yuan H., Ma Y., Li S., Zang Z., Zhang J. (2024). Identification and Characterization of MADS-Box Gene Family in Flax, *Linum usitatissimum* L. and Its Role under Abiotic Stress. Iscience.

[93] Lu J., Wang Z., Li J., Zhao Q., Qi F., Wang F., Xiaoyang C., Tan G., Wu H., Deyholos M.K. (2023). Genome-Wide Analysis of Flax (*Linum usitatissimum* L.) Growth-Regulating Factor (GRF) Transcription Factors. Int. J. Mol. Sci..

[94] Ye J., Yang K., Geng W., Chen K., Yao Z., Zhang W., Zheng J., Xu F. (2025). A Novel miR396-GbMYB41 Module Regulates Flavonoid Biosynthesis in *Ginkgo biloba*. Ind. Crops Prod..

[95] Liebsch D., Palatnik J.F. (2020). MicroRNA miR396, GRF Transcription Factors and GIF Co-Regulators: A Conserved Plant Growth Regulatory Module with Potential for Breeding and Biotechnology. Curr. Opin. Plant Biol..

